# Variation in denitrifying bacterial communities along a primary succession in the Hailuogou Glacier retreat area, China

**DOI:** 10.7717/peerj.7356

**Published:** 2019-08-14

**Authors:** Yan Bai, Xiying Huang, Xiangrui Zhou, Quanju Xiang, Ke Zhao, Xiumei Yu, Qiang Chen, Hao Jiang, Tashi Nyima, Xue Gao, Yunfu Gu

**Affiliations:** 1 Department of Microbiology/ College of Resources/Sichuan Agricultural University, Sichuan Agricultural University, Chengdu, Sichuan, China; 2 Institute of Mountain Hazards and Environment, Chinese Academy of Sciences, Chengdu, Sichuan, China; 3 Institute of Agricultural Resources and Environmental Science, Tibet Academy of Agricultural and Animal Husbandry Sciences, Lhasa, Tibet, China

**Keywords:** Hailuogou Glacier retreat area, Primary succession, Denitrifying bacteria, Community, Abundance

## Abstract

**Background:**

The Hailuogou Glacier is located at the Gongga Mountain on the southeastern edge of the Tibetan Plateau, and has retreated continuously as a result of global warming. The retreat of the Hailuogou Glacier has left behind a primary succession along soil chronosequences. Hailuogou Glacier’s retreated area provides an excellent living environment for the colonization of microbes and plants, making it an ideal model to explore plant successions, microbial communities, and the interaction of plants and microbes during the colonization process. However, to date, the density of the nitrogen cycling microbial communities remain unknown, especially for denitrifiers in the primary succession of the Hailuogou Glacier. Therefore, we investigated the structural succession and its driving factors for denitrifying bacterial communities during the four successional stages (0, 20, 40, and 60 years).

**Methods:**

The diversity, community composition, and abundance of *nosZ*-denitrifiers were determined using molecular tools, including terminal restriction fragment length polymorphism and quantitative polymerase chain reactions (qPCR).

**Results:**

*nosZ*-denitrifiers were more abundant and diverse in soils from successional years 20–60 compared to 0–5 years, and was highest in Site3 (40 years). The denitrifying bacterial community composition was more complex in older soils (40–60 years) than in younger soils (≤20 years). The terminal restriction fragments (T-RFs) of *Azospirillum* (90 bp) and *Rubrivivax* (95 bp) were dominant in soisl during early successional stages (0–20 years) and in the mature phase (40–60 years), respectively. Specific T-RFs of *Bradyrhizobium* (100 bp) and *Pseudomonas* (275 bp) were detected only in Site3 and Site4, respectively. Moreover, the unidentified 175 bp T-RFs was detected only in Site3. Of the abiotic factors that were measured in this study, soil available phosphorus, available potassium and denitrifying enzyme activity (DEA) correlated significantly with the community composition of *nosZ*-denitrifiers (*P* < 0.05 by Monte Carlo permutation test within RDA analysis).

## Introduction

Global warming has accelerated the retreat of glaciers, which has resulted in the exposure of soil and rocks that were once covered in ice (Schmidt et al., 2016). In this nitrogen (N) poor environment, microbes are usually the first colonizers and keystone players, especially in many pristine environments, including glacier retreat areas (Bradley et al., 2016). Microbes are closely associated with plant successions during the colonization process, and the plants influence the bacterial succession during pedogenesis (Brown & Jumpponen, 2014). Furthermore, under the nutrient-limited conditions, microbes can also promote plant growth by secreting extracellular enzymes to hydrolyze polymers and release organic monomers (Schimel & Bennett, 2004; Schimel & Weintraub, 2003). Thus, soil microorganisms are crucial to the succession of plant communities, as well as the transformation and cycling of soil nutrients (Jiang et al., 2019). Previous studies have shown that the composition of soil microbial communities exhibit distinct patterns at different succession stages along retreating glaciers (Knelman et al., 2012; Schmidt et al., 2016; Insam et al., 2017). For example, at the Rotmoosferner Glacier, Austria, the successional age of the glacier forefield shaped the nitrogen cycling processes and microbial community composition (Nicol, 2010). Furthermore, Jiang et al. (2018) found that the successional trajectories and driving forces for soil microbial communities were different, where microbial community structure was shaped by edaphic properties during the early stages (3–52 years), and by vegetation properties during the later stages (80–120 years), highlighting the importance of soil-plant-microbe interactions during ecosystem successions.

Denitrification is one of the metabolic pathways belonging to the nitrogen cycle. This pathway converts nitrate into nitrite, nitric oxide (NO), nitrous oxide (N_2_O) or nitrogen gas (N_2_) (Lashof & Ahuja, 1990). N_2_O, and N_2_ are gases, and are not readily available to support microbial growth; therefore, they are typically released to the atmosphere. N_2_ constitutes over 70% of atmospheric gases, thus the release of N_2_ to the atmosphere is benign (Lashof & Ahuja, 1990). However, N_2_O is emitted at a level 300 times higher than CO_2_, has important implications in terms of global warming and the ecological functioning of natural ecosystems (Howarth, 2004; Solomon et al., 2007; Wuebbles, 2009). The reduction of N_2_O to N_2_ is the last step in the denitrification pathway, which is catalyzed by N_2_O reductase, which is encoded by *nosZ* (Zumft, 1997; Hallin et al., 2018), and N_2_O reductase is the most sensitive enzyme to environmental conditions (Šimek & Cooper, 2002). Therefore *nosZ* is used as a molecular marker to detect the presence of denitrifying microorganisms (Philippot, 2002). Wang et al. (2012) have shown that the abundance of *nosZ* in a microbial community leads to reduction in N_2_O in the surface soils and soil core. The abundance of *nosZ* has been used to reflect variation of denitrifying bacterial communities throughout primary succession in a glacier retreat (Kandeler et al., 2006). In the Damma glacier, Switzerland, *nosZ* copy numbers were lowest during the early successional stages of a glacier retreat, and significantly correlated with the denitrifying enzyme activities (Brankatschk et al., 2011). However, these processes were not correlated with the abundance of nitrite reductases (*Nir*) that are encoded by cytochrome cd 1-containing (*nirS*) and copper (*nirK*). In another study, the amount of organic substances was the most important factor in determining the abundance of *nosZ* denitrifiers during the primary succession of Rotmoosferner glacier foreland (Kandeler et al., 2006). These studies revealed that the variation and driving forces of the community composition of *nosZ*-denitrifiers along a glacier forefield chronosequence. To date, little is known about the abundance and community composition of the *nosZ*-denitrifiers along the Hailuogou glacier foreland. Thus studying the denitrifying bacterial community, with a focus on *nosZ* denitrifying bacteria, on the Hailuogou glacier foreland will provide a theoretical foundation of nitrogen cycle in this area.

The Hailuogou Glacier, located on the Gongga Mountain, has been retreating continuously since the end of the Little Ice Age (LIA) (Li et al., 2018), and is an excellent study site to understand soil development and the succession of microbial and plant communities. The Gongga Mountain’s mild and humid climate allows for rapid moraine colonization by soil microorganisms and plants that promote fast ecosystem development. In addition, there is a complete primary succession series from bare land to a lush forest stage along the approximately 2-km-long belt. Previous studies in this area have investigated specific processes, such as pedogenesis (He & Tang, 2008; Zhou et al., 2013) and plant successions (Yang et al., 2014). Recently, the roles of microbial communities as driving forces of ecosystem formation along glacier forefield chronosequences have been demonstrated in other areas (Sun et al., 2016; Jiang et al., 2019; Jiang et al., 2018). However, it is unclear whether the microbial community played a role in the denitrifying process in the primary succession of the Hailuogou Glacier.

In this study, the diversity and abundance of *nosZ* denitrifying bacterial communities in four successional stages, time of exposure after the glacier melted for 0, 20, 40, and 60 years, were assessed by chemical analysis, quantitative PCR, and terminal restriction fragment length polymorphism. The objectives of this study were (i) to investigate the variation in the *nosZ* denitrifying bacterial community in the four different successional stages and (ii) to understand the relationships between the *nosZ* denitrifying bacterial community and soil properties. We hypothesized that the variation in the activity and composition of the *nosZ* denitrifying bacterial community would be strongly correlated with the successional stages.

## Material and Methods

### Study area

The Hailuogou Glacier (29°34′07.83″N, 101°59′40.74″E, Sichuan Province, China) is on the eastern slope of Gongga Mountain, which is on the southeastern edge of the Tibetan plateau in southwest China. Against the background of a warming climate, the Hailuogou Glacier is shrinking continuously and melting rapidly. This glacier retreat area currently extends to the northeast and has a horizontal length of two km, with an elevation difference of 100 m. In this area, the climate is characterized by an average annual precipitation of 1,947 mm, and most rainfall occurrs between June and October (Lei et al., 2015). There are considerable seasonal temperature fluctuations, ranging from −4.3 °C in January to 11.9 °C in July, with an annual mean temperature of 3.8 °C. The relatively mild and humid climate of Hailuogou glacier promotes ecosystem development and rapid colonization by plants. Along the approximately 2-km-long belt of the Hailuogou glacier foreland, there is a complete primary succession series from bare land to climax vegetation communities (Mapelli et al., 2018; Jiang et al., 2018). In the Hailuogou glacier retreated area where the ice melted about 60 years ago, a community resembling a mature phase community has been established, dominated by *P.brachytyla* and *A. fabri* (Zhou et al., 2018). Primary succession was marked by shifts in dominant species as follows: (i) *Astragalus* spp*. Hippophae rhamnoides L.* and *Salix magnifica* during the 5- to 12-year stage, (ii) *H. rhamnoides, Salix* spp*.* and *Populus purdomii* during about 35 years, (iii) *Betula utilis, P. purdomii, Abies fabri* at the 52-year stage, (iv) *Betula utilis, P. purdomii,* and *A. fabri* becoming replaced by *A. fabri*, with high biomass productivity of the plant community at the 80-year stage, and (v) *P. brachytyla* and *A. fabri* at the 120-year phase (Zhou et al., 2018). The soil parent material is mainly composed of biotite schist, granodiorite, and quartzite, with small amounts of phyllite, slate, and chlorite schist (Zhou et al., 2013). The different sampling points are listed in Table 1.

**Table 1:** Details of the sampling sites within the Hailuogou Glacier retreat area.

Site code	Successional time (years)	Longitude	Latitude	Horizontal distance (m)	Elevation (m)
Site1	≤5	101°59′32.8″	29°34′03.6″	100	2,956
Site2	≤20	101°59′40.2″	29°34′04.1″	300	2,948
Site3	≤40	101°59′43.7″	29°34′05.7″	600	2,940
Site4	≤60	101°59′48.9″	29°34′07.9″	1,200	2,926

**Notes.**

Horizontal distance refers to the distance from the glacier.

Site1 Soil successional ages ≤5 years Site2 Soil successional ages ≤20 years Site3 Soil successional ages ≤40 years Site4 Soil successional ages ≤60 years

### Soil sampling

**Figure 1:**
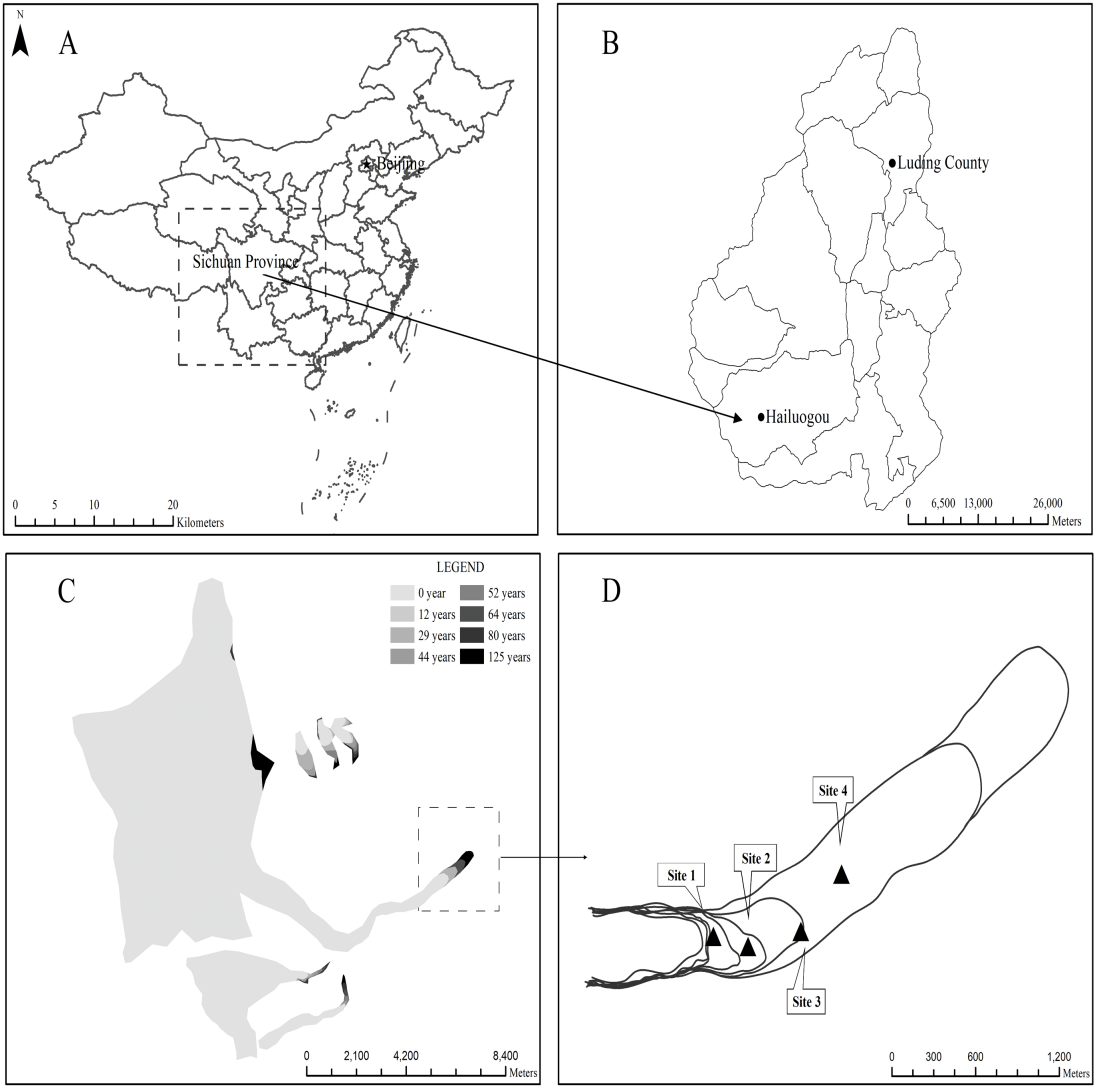
Map of soil samples collected from sites representing a chronosequence of primary succession in the Hailuogou Glacier area. The location of Hailuogou glacier in China map and Sichuan map (A, B), changes in Hailuogou glacier during the different primary successional stages (C) and soil samples collected from different primary successional sites in the Hailuogou glacier area, which a black triangle (▴) represent the sampling sites in the present study (D). Site1: Soil successional ages ≤5 years; Site2: Soil successional ages ≤20 years; Site3: Soil successional ages ≤40 years; Site4: Soil successional ages ≤60 years.

Soil samples were collected from four sites, representing four successional stages at 0, 20, 40, and 60 years after melting of the glacier (Fig. 1). Site1 is a bare land at the bare-dwarf vegetation stage with the pioneer species *Astragalus* spp.*,* and the successional time is below 5 years. Site2 represents successional stage 20 years, and the horizontal distance from the end of the glacier is about 300 m. In Site2, *H. rhamnoides L.* and *Salix magnifica* are the dominant species. Site3 is at successional time 40 years, and the horizontal distance from the end of the glacier is about 600 m, with dominant species *H. rhamnoides, Salix* spp. and *P. purdomii* Site4 is at succession age 60 years, and the horizontal distance from the end of the glacier is about 1,200 m (Table 1). The dominant plant species of Site4 are *P. purdomii* and *Betula utilis*, and some species of pine (*A. fabri*). To avoid erosion and deposition effects, all sampling plots were located in the middle of gentle slopes, far from the small streams inside the primary succession areas. Moreover, all plots (except those in the Site1) were placed under the canopies of the dominant plant species.

Soil samples were collected from four sites with different lengths of exposure time (0, 20, 40 and 60 years) (Fig. 1). For each site, we sampled five sampling plots of at least 20 × 20 m^2^, with a minimum distance of 10 m between plots. From each plot, five soil cores to a depth of 20 cm were collected from the center and the corners of the plot using a 6.5-cm diameter soil corer after removing surface litter. The samples were mixed to obtain one composite sample for each plot. Subsequently, the samples were stored in plastic bags on ice and divided into two portions: one was air-dried and sieved through a 2-mm mesh screen for physicochemical analyses, and the other one was stored at −80 °C for soil total DNA extraction.

### Soil properties and denitrifying enzymatic activity analysis

Soil pH was determined using a potentiometer pH meter (Rex Electric Chemical PHSJ-3F). Soil total nitrogen (TN) was measured using the semi-micro Kjeldahl method (Bremner & Mulvaney, 1996), and soil organic carbon (SOC) was determined using the potassium dichromate oxidation (Bao, 2007). Soil available phosphorus (AP) and available potassium (AK) were assessed via the sodium bicarbonate extraction-Mo-Sb colorimetry methods and ammonium acetate extraction-flame photometry, respectively (Watanabe et al., 2017; Bao, 2007). DEA was determined using the C_2_H_2_ inhibition method. Ten grams of each soil sample were placed in 150 mL plasma flasks containing 10 mL of nitrate solution (100 mg N L^−1^). The flasks were then sealed and purged three times, by vacuuming for 20 min and flushing with helium. They were then incubated either with 2.5% (v/v) of C_2_H_2_ to determine the denitrifying enzyme activity or without C_2_H_2_ to estimate the N_2_O production. The N_2_O concentrations, with or without acetylene, were monitored with a gas chromatograph (GC-2010PLUS; Shimadzu, Kyoto, Japan) (Dambreville et al., 2006).

### Total soil DNA extraction

Total soil DNA was extracted from 0.5 g of fresh soil using a FastDNA SPIN Kit for Soil (MP Biomedicals, Solon, OH, USA) following the manufacturer’s protocol. The concentration and quality of extracted DNA were measured with a Nano-200 spectrophotometer (Aosheng, Hangzhou, China), and DNA samples were stored at −20 °C for further analysis.

### Quantitative PCR

The standard plasmid for real-time PCR (qPCR) was made by a sequenced clone with known gene copy number and a series of 10-fold dilutions for the standard curves. The qPCR assays were performed on an ABI 7500 Sequence detection system (Applied Bio-systems, CA) using SYBR green method. Three replicate reactions were set up for each sample, and each reaction was performed in a total of 25 µL volume including12.5 µL of ABI Power SybrGreen qPCR Master Mix (Applied Biosystems, Foster City, CA, USA), 1 µM (each) primer, 1.25 µL of template DNA, adjusted to a concentration of 20 ng µL ^−1^; sterile distilled water was used to bring the final volume up to 25 µL. The primer pairs *nosZ* 2F (5′-CGYTGTTCMTCGACAGCCAG-3′) and *nosZ* 2R (5′-CGSACCTTSTTGCCSTYGCG-3′) were used to quantify *nosZ* clade I (Dini-Andreote et al., 2016). The qPCR program consisted of an initial denaturation step at 95 °C for 10 min, 40 cycles of 95 °C for 15 s, 60 °C for 60 s, and 72 °C for 30 s. The *R*^2^ value (amplification efficiency) was 0.97 (62.6%) for *nosZ*.

### PCR amplification of *nosZ* fragments and T-RFLP

Terminal restriction fragment length polymorphism (T-RFLP) analysis of *nosZ* was carried out using the primer pair *nosZ-* F (5′-CGCRACGGCAASAAGGTSMSSGT-3′) and *nosZ* 1622R (5′-CAKRTGCAKSGCRTGGCAGAA-3′), where the forward primer was labeled with 6-carboxyfluoroscein (FAM) (Wang et al., 2017). The PCR reaction mixture volume was 50 µL, and consisted of 60 ng of template DNA, 0.4 µM (each) primer, 25 µL of PCR master mix (Tiangen, Beijing, China), and sterile distilled water to obtain a final volume of 50 µL. The PCR was performed with the following thermal profile: 95 °C for 3 min, followed by 30 cycles of 95 °C for 30 s, 58 °C for 1 min, and 72 °C for 1 min, with a final extension step at 72 °C for 5 min. The PCR products were checked using 1.0% agarose gels electrophoresis and visualized with a UV Transilluminator Model M-26 (UVP, USA) after ethidium bromide staining. The amplified *nosZ* fragments were digested with *BstuI* and *HhaI* endonucleases (NEB, Ipswich, MA, USA) at 37 °C for 6 h, followed by further purification with a Tiangen DNA purification kit (Tiangen, China). The T-RFLP profiles were then generated by capillary electrophoresis using an ABI Prism 3100 Genetic Analyzer at Sangong Corporation (Shanghai, China).

T-RFLP data analysis was performed using the methods described by Ulrich & Becker (2006). Sizes and relative abundances of terminal restriction fragments (T-RFs) were performed using the software package Peak Scanner version 1.0 (Applied Biosystems, Inc.). The peak heights of T-RFs with size differences of ≤2 bp in an individual profile were combined and considered to be one fragment (Zhang et al., 2017). The T-RFs with a relative abundance <1% were excluded from further analysis (Zhang et al., 2017), and fragments between 60 and 640 bp corresponding to the size range of the standard were incorporated for further analysis (Egert & Friedrich, 2003). Three different measures of diversity, the Shannon–Weiner index (H′), richness (*d*), and evenness (*e*), were calculated using the formulas from Bharti et al. (2015) for each T-RFLP pattern.

### Statistical analysis

All results were based on five replicate treatment plots. The soil physicochemical parameters and *nosZ* diversity were analysed by analysis of variance (ANOVA) using SPSS 21.0 Software (SPSS, Chicago, IL, USA). The differences of soil physicochemical parameters and *nosZ* diversity were performed using Duncan’s new multiple-range test of one-way ANOVA at 5% level of significance with SPSS 21.0. Using the vegan package in R. Principal components analysis (PCA) was employed to further examine the variation in soil parameters among different successional stages, and analysis of similarity (ANOSIM) was used to test significant differences among sites. Pearson’s correlation analysis was conducted to examine the relationships between soil properties and *nosZ* diversity using the software package SPSS 21.0. To further investigate the effect of edaphic properties on the *nosZ*-denitrifier community profiles, redundancy analysis (RDA) with the software package CANOCO 5.0 was conducted. Before the RDA analysis, a detrended correspondence analysis for the specific microbial groups was performed to confirm that the linear ordination method was appropriate for the analyses (gradient lengths < 3).

## Results

### Soil properties and denitrifying activities in different successional stages

All physicochemical properties examined responded significantly to four successional stages (Site1, ≤5 years; Site2, ≤20 years; Site3, ≤40 years; and Site4, ≤60 years) (Table 2). Soil pH displayed a gradual decrease as a function of time, from 8.42 (site1) to 7.19 (Site3). TN and SOC continuously accumulated from Site1 to Site4. AP content and DEA concentration remained at a low level during the initial stage, and then sharply increased to its highest value at Site3 but fluctuated at Site4 (Table 2, Dataset S1). AK levels increased significantly throughout the early three stages but decreased significantly in Site4 (Table 2, Dataset S1).

**Table 2:** Soil properties of the different sampling sites.

Sampling code	pH	SOC (g/kg)	TN (g/kg)	AP (mg/kg)	AK (mg/kg)	DEA (µg/g/h)
Site1	8.41 ± 0.08a	4.87 ± 0.14d	0.286 ± 0.01d	0.980 ± 0.07c	13.60 ± 0.45d	0.076 ± 0.00d
Site2	7.85 ± 0.03b	9.86 ± 0.35c	0.463 ± 0.02c	1.24 ± 0.04c	40.45 ± 1.78c	0.098 ± 0.01c
Site3	7.31 ± 0.05c	16.19 ± 0.63b	0.765 ± 0.04b	5.54 ± 0.34a	120.80 ± 3.36a	0.311 ± 0.01a
Site4	7.19 ± 0.15c	30.77 ± 1.93a	1.11 ± 0.09a	3.70 ± 0.15b	73.59 ± 2.23b	0.128 ± 0.00b

**Notes.**

Values represent mean ± standard error (*n* = 5). Values within the same column followed by the same letter do not differ at *P* < 0.05.

SOC Soil organic carbon TN Total nitrogen AP Available phosphorus AK Available potassium DEA Denitrifying enzyme activity Site1 Soil successional ages ≤5 years Site2 Soil successional ages ≤20 years Site3 Soil successional ages ≤40 years Site4 Soil successional ages ≤60 years

**Figure 2:**
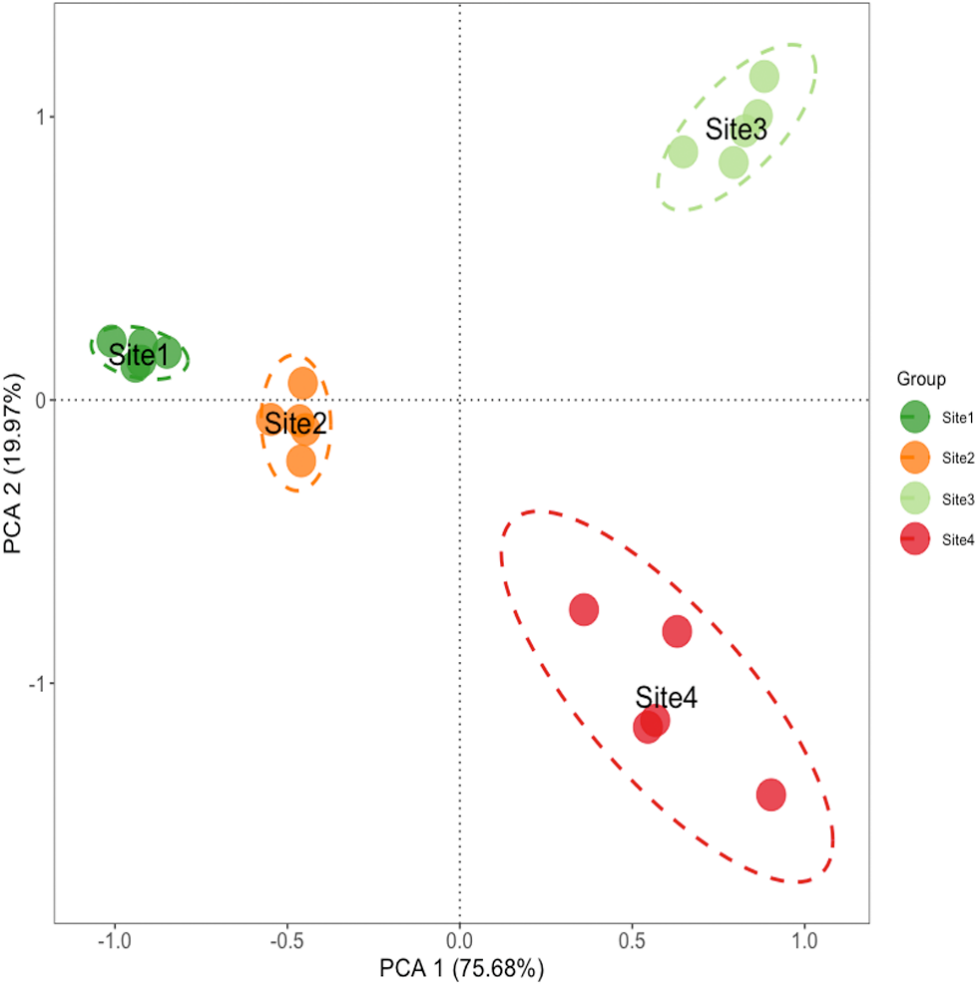
Principal component analysis of soil properties in different soil successional stages. Principal components analysis of soil properties in different soil successional stages. Site1: Soil successional ages ≤5 years; Site2: Soil successional ages ≤20 years; Site3: Soil successional ages ≤40 years; Site4: Soil successional ages ≤60 years.

Principal component analysis (PCA) showed clear separation of the four successional sites indicating that primary succession resulted in distinct microhabitats (ANOSIM: *R* > 0.9, *P* < 0.05) (Fig. 2, Dataset S1). Samples generally grouped by soil properties across axis 1, which described 75.68% of variation. Axis 2 described 19.97% of variation (Fig. 2, Dataset S1). These results suggest that nutrient levels are changing significantly along the soil chronosequence.

**Figure 3:**
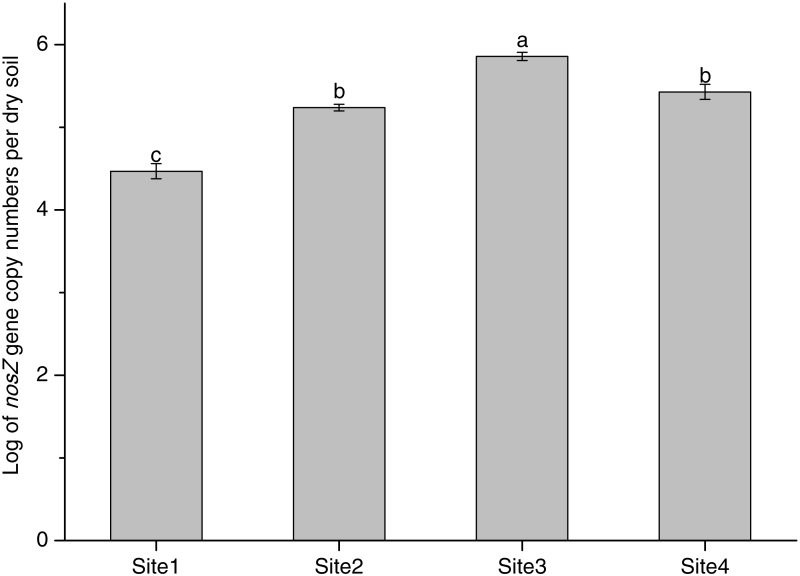
The *nosZ* copy numbers of different soil successional stages. Site1: Soil successional ages ≤5 years; Site2: Soil successional ages ≤20 years; Site3: Soil successional ages ≤40 years; Site4: Soil successional ages ≤60 years. Different lowercase letters indicate significant differences between the nosZ gene copy numbers of different soil successional stages (*P* < 0.05). All data were expressed as the average of five replicates ±standard error (SE).

### The *nosZ* denitrifying bacterial community abundance and diversity in different successional stages

The abundance of *nosZ* increased significantly from Site1 (4.47 ± 0.09 log copy numbers g^−1^ dry soil) to Site3 (5.86 ± 0.05 log copy numbers g^−1^ dry soil), and decreased significantly in Site4 (5.43 ± 0.09 log copy numbers g^−1^ dry soil) (Fig. 3, Dataset S2). These results highlight that the densities of denitrifying microorganisms significantly changed along a glacier forefield chronosequence.

The diversity varied significantly among the four successional stages (*P* < 0.05) (Table 3). Shannon–Weiner index and richness of *nosZ* containing communities increased continuously in the three earlier stages but decreased significantly in the last stage. Site3 had the highest values of Shannon–Weiner index (2.54 ± 0.08) and richness (18.41 ± 0.08). Evenness increased gradually throughout the primary succession, with the highest values observed in Site4 (1.30 ± 0.04) (Table 3, Dataset S3).

**Table 3:** The *nosZ* gene diversity along successional stages.

Sample codes	Shannon–Weiner index (H′)	Richness *(d)*	Evenness *(e)*
Site1	1.82 ± 0.02c	8.99 ± 0.44b	1.03 ± 0.01b
Site2	1.87 ± 0.01c	10.04 ± 0.07b	1.13 ± 0.05b
Site3	2.54 ± 0.08a	18.41 ± 0.08a	1.28 ± 0.01a
Site4	2.32 ± 0.09b	11.57 ± 0.53b	1.30 ± 0.04a

**Notes.**

Values represent mean ± standard error (*n* = 5). Values within the same column followed by the same letter do not differ at *P* < 0.05.

Site1 Soil successional ages ≤5 years Site2 Soil successional ages ≤20 years Site3 Soil successional ages ≤40 years Site4 Soil successional ages ≤60 years

Pearson’s correlation analysis revealed that Shannon–Weiner index was significantly linked to soil AP (*P* < 0.01) and AK (*P* < 0.05), while richness was positively correlated with AP, AK, and DEA (*P* < 0.05). Evenness was negatively correlated with soil pH (*P* < 0.01) (Table 4, Dataset S4). The abundance of *nosZ* was significantly correlated with DEA, richness (*P* < 0.01) and AK (*P* < 0.05).

**Table 4:** Pearson’s correlations between soil properties, denitrifying enzyme activity, and *nosZ* gene diversity.

	pH	SOC	TN	AP	AK	DEA	Abundance
Shannon–Weiner index (H′)	−0.879	0.684	0.776	.995^**^	.968^*^	0.871	0.901
Richness (*d*)	−0.658	0.292	0.428	0.932	.953^*^	.999^**^	0.995^**^
Evenness (*e*)	−.995^**^	0.879	0.944	0.872	0.872	0.647	0.728
Abundance	−0.709	0.335	0.473	0.935	.970^*^	.990^**^	1

**Notes.**

SOC Soil organic carbon TN Total nitrogen AP Available phosphorus AK Available potassium DEA Denitrifying enzyme activity Abundance The *nosZ*-denitrifying bacterial abundance

** *P* < 0.01

* *P* < 0.05

### The *nosZ* denitrifying bacterial community composition in different successional stages

**Figure 4:**
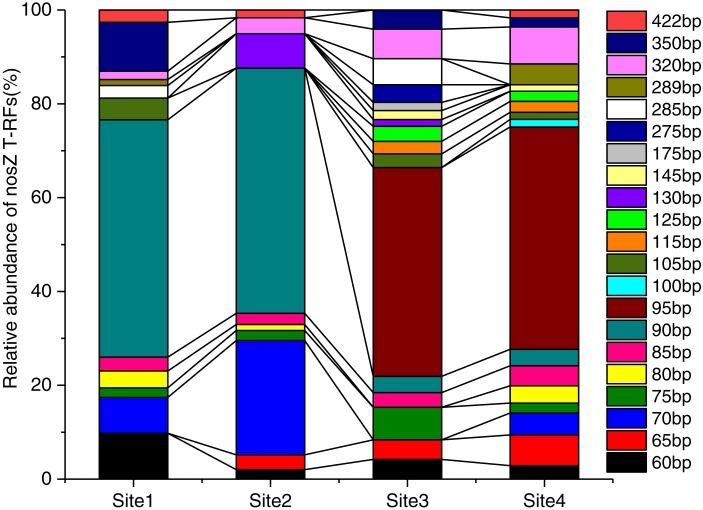
The community composition of *nosZ*-denitrifiers at different soil successional stages. The relative abundances of nosZ terminal restriction fragments (T-RFs) in different successional stages. The relative abundance of T-RFs is given as a percentage of the total peak height. Fragment sizes within the graph indicate the sizes (bp) of the T-RFs in T-RFLP. Site1: Soil successional ages ≤5 years; Site2: Soil successional ages ≤20 years; Site3: Soil successional ages ≤40 years; Site4: Soil successional ages ≤60 years.

The varied T-RFs patterns of the different successional stages could be used to characterize community structure and changes in the diversity of *nosZ*-denitrifiers. The soils of four successional stages had some variations in T-RFs patterns of *nosZ* and the relative abundances of different T-RFs were observed (Fig. 4, Dataset S5). In the *nosZ* T-RFs profiles, dominant T-RFs of 60, 90, and 350 bp were observed in Site1, dominant T-RFs of 70, 90, and 130 bp were observed in Site2, and the predominant fragment was T-RF of 90 bp for both sites (48.3% and 48.9% for Site1 and Site2, respectively). However, Site3 and Site4 had the predominant T-RF of 95 bp, and this fragment was only found in Site3 and Site4 (37.9% and 43.7% for Site3 and Site4, respectively). There were also non-overlapping T-RFs that were less abundant in the different sites. For example, T-RFs of 100, 115, 125, 145 and 275 bp were only present in Site4 and Site3.

In the combined *in silico* T-RFLP analysis of *nosZ* sequences, *nosZ* (Accession# KT329435) sequence from the NCBI database was used as the reference sequence. The dominant 90 bp T-RF was consistent with the T-RF of *Azospirillum*, the 95 bp T-RF was consistent with *Rubrivivax*, and the T-RFs of 100, 145, and 275 bp were consistent with *Bradyrhizobium*, *Ochrobactrum*, and *Pseudomonas*, respectively, and some other T-RFs belonged to unidentified genus (Fig. 4, Dataset S5).

### Redundancy analysis of *nosZ* denitrifying bacterial community composition and soil properties

**Figure 5:**
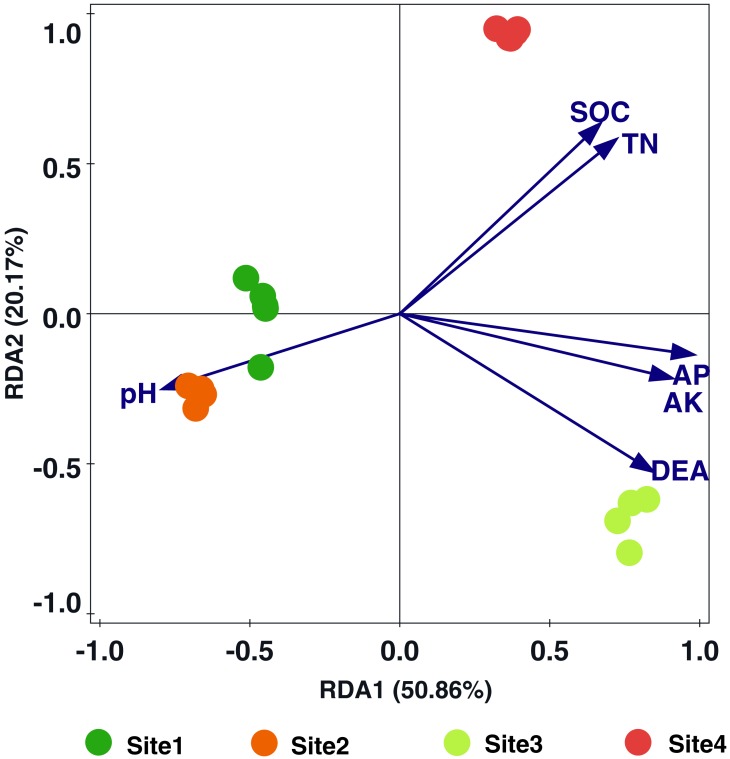
Redundancy analysis ordination diagram of the *nosZ*-denitrifying bacterial community composition associated with environmental variables. The relative abundances of nosZ terminal restriction fragments (T-RFs) in different successional stages. The relative abundance of T-RFs is given as a percentage of the total peak height. Fragment sizes within the graph indicate the sizes (bp) of the T-RFs in T-RFLP. Site1: Soil successional ages ≤5 years; Site2: Soil successional ages ≤20 years; Site3: Soil successional ages ≤40 years; Site4: Soil successional ages ≤60 years.

The relationships between soil properties and *nosZ* denitrifying bacterial community were analyzed using a redundancy analysis (RDA) (Fig. 5, Dataset S6). The *nosZ* denitrifying bacterial community structure were differentiated into three clusters: Site1-Site2 (cluster 1), Site3 (cluster 2), and Site4 (cluster 3) (Fig. 5). Furthermore, among the environmental factors, AP, AK, and DEA were significantly correlated with *nosZ*-denitrifiers composition during the primary succession of the Hailuogou Glacier (*P* < 0.05 by Monte Carlo permutation test within RDA analysis), whereas no significant correlation was observed between *nosZ*-denitrifiers and other soil characteristics.

## Discussion

### Soil properties at different successional stages

Soil development is dependent upon various soil forming factors, such as parent material, topography, climate, biota, and time, and how these factors fluctuate and interact (Brady & Weil, 2004). We found that soil properties changed significantly with distance from the Hailuogou glacier. Based on the studied parameters, soil pH was highest and nitrogen and available phosphorus resources were most limited during the initial stage of ≤5 years from the melting of the glacier. This stage was characterized by coarse and bare soil, with only small areas of the surface being covered by moss. The decrease in the range of topsoil pH that we observed in the 60 years succession of the Hailuogou glacier are similar to the decrease of soil pH (about 1 unit) of the Damma glacier forefield over 140 years (Dümig, Smittenberg & Kogel-Knabner, 2011). The decrease of pH can be ascribed to increased excretion of organic acids by plant roots and accumulation of acid-forming soil organic matter with progressive vegetation development (Lauber et al., 2009; Zhou et al., 2013). Previous studies showed that a greater variety of plant litter entering the soil as the succession proceeded correlated with accelerated organic substrates and nitrogen accumulation (He & Tang, 2008; Mavris et al., 2010), and this may explain our findings that SOC and TN contents increased significantly along the successional 60 years. Furthermore, the humid and mild climate on the Hailuogou Glacier chronosequence is more favorable for rapid accumulation of organic carbon (Lei et al., 2015). In our study, AP and AK content was maintained at a low level during the initial stages, and sharply increased at Site3. The high concentration of AP during the later phase was likely a consequence that from the 40 to 60 year-old sites, the decreased pH (7.19) resulted in intensified bedrock weathering, increased liberation of formerly occluded bedrock P, and its conversion into available P compared to the younger soils (0–20 years) (Lajtha & Schlesinger, 1988). Topsoil AP concentration increased markedly at 40 years of deglaciation, and subsequently, the abundant annual precipitation (approximately 1,947 mm) led to a strong erosion and loss of soil P (Jiang et al., 2018). Our findings suggest that these sequences of events led to the decrease in available P after >40 years of soil development.

### Denitrifying enzyme activity in different successional stages

While denitrification is a critical part of the nitrogen cycle, how denitrification develops in soil over time remains unclear. In general, potential enzyme activities related to dry soil increases along the chronosequence (Brankatschk et al., 2011). A previous study showed that potential denitrification activity increased gradually along a chronosequence of a glacier forefield (Brankatschk et al., 2011). Salles et al. (2017) found that the potential denitrification activity increased progressively along a salt marsh succession, and reached extreme values at the successional 100 years. In our study, DEA remained low level during the initial stage of the glacier forefield, and sharply increased to its highest value at successional 40 years. At well-developed soil sites, plant root exudation can enhance water retention and reduce oxygen diffusion. These mechanisms can enhance thesoil denitrification rates, which may explain the results of our study (Kandeler et al., 2006). Furthermore, denitrification is determined by physical, chemical and biological factors, including soil moisture, soil carbon, and soil nitrogen (Leloup et al., 2018). Most studies have found that soil carbon and pH have the fundamental effect on denitrification (Zeng et al., 2015). Specifically, soil denitrifying activity is often correlated with soil pH, soil organic carbon, nitrate, and soil moisture (Salles et al., 2017). However, soil microbial denitrifiers are the primary organisms that drive denitrification. Petersen et al. (2012) found that the abundance of *nirK/S* had indirect effects on potential denitrification activity. In contrast, Brankatschk et al. (2011) found that potential enzyme activities correlated with *nosZ* gene copy number, and not with the abundance of *nirS* or *nirK*. In our study, DEA was not correlated with soil properties, but showed significant correlations with the abundance of *nosZ-* denitrifiers. The strong correlation between *nosZ* gene abundance and enzymatic activities may explain why N_2_O reducers occur in many different groups of microorganisms with different physiological backgrounds. However, induction of the N_2_O reductase is linked to the presence of nitrate, thus similarly regulated for all groups of N_2_O reducers (Zumft, 1997; Hallin et al., 2009).

### The *nosZ* denitrifying bacterial community abundance response to different successional stages

Denitrifying bacteria are widely distributed in different ecosystems and strongly shaped by the ecological conditions. For example, soil depth and seasonal changes significantly affect the composition and abundance of denitrifying bacterial communities (Henry et al., 2006; Wu et al., 2017; Tang et al., 2016). The abundance of functional genes (*nirS/K, nosZ*) could also indirectly reflect the denitrification activity of the soil (Wu et al., 2017). Therefore, quantitative analysis of denitrifying functional genes is often used to study changes in denitrification in a given ecosystem. *nosZ* is part of a well-known clade of nitrous oxide reductases (clade I). While *nosZ* belonging to clade II have been recently discovered (Sanford et al., 2012; Jones et al., 2013), we only quantified *nosZ* clade I genes. A study on similar soil stages at the Schiermonikoog Island (Dini-Andreote et al., 2016) quantified both clades, and observed that the abundance of clade I was ca. 10-fold higher than clade II across all successional stages. Thus, while we did not quantify *nosZ* clade II genes, we believe that our results reflect the most abundant denitrifying bacteria along the chronosequence. Henry et al. (2006) found that *nosZ* abundance gradually increased and peaked at the early successional stages, with subsequent decreases during the mature stage. Similar to this, we found that *nosZ* abundance increased significantly from the initial (Site1, 4.47 ± 0.09 log copy numbers g^−1^ dry soil) to the later stages (Site3, 5.86 ± 0.05 log copy numbers g^−1^ dry soil), then decreased significantly afterwards (Site4, 5.43 ± 0.09 log copy numbers g^−1^ dry soil) (Fig. 3). As nitrous oxide reductase is sensitive to environmental variation changes in *nosZ* abundance likely caused by the differences in plant development, soil formation and the accumulation of soil nutrients in the different successional stages (Zhang et al., 2017). For example, Meng et al. (2017) showed that the survival and growth of soil microorganisms are facilitated by the abundance of plant species and plant litter, leading to a gradual increase in functional genes throughout the succession. In addition, Salles et al. (2017) indicated the high soil pH of salt marshes is the most limiting factor for microbial growth, and *nosZ* abundance remained constant throughout the succession of such ecosystems.

### Variation in *nosZ* denitrifying bacterial communities in response to primary succession

Denitrification in soils is often rate-limited by the last step, the conversion of nitrous oxide to nitrogen, which is driven by *nosZ*-denitrifiers (Philippot, 2002; Kandeler et al., 2006). However, the consequences of such modifications of the communities composition and relative abundances of the denitrification genes for N_2_O and N_2_ fluxes in primary succession ecosystem are still unclear (Kandeler et al., 2006). Thus, one of the main purposes of this study was to monitor the response of the *nosZ*-denitrifier community structure to primary succession of Hailuogou glacier foreland. A previous study revealed that *nosZ* denitrifiers presented clear trends of succession during soil development (Zeng et al., 2016). The abundance of *nosZ* gene was lowest in young soil (<10 years), and increased significantly with progressing succession, with a maximum in late succession (Kandeler et al., 2006; Brankatschk et al., 2011). In our study, specific and dominant *nosZ* T-RFs were changed during the primary succession, and different sites had different proportions in the relative abundance of T-RFs. For example, we detected 90 bp T-RFs, which was affiliated with *Azospirillum*, and dominated in soil during early successional stages (0–20 years)*.* However, T-RFs of 95 bp was dominant in the mature phase (40–60 years), and this fragmentc *Rubrivivax*. Moreover, some less abundant T-RFs fragments, such as 100 and 275 bp T-RFs were observed only in Site3 and Site4, which were affiliated with *Bradyrhizobium* and *Pseudomonas*, respectively, and the unidentified 175 bp T-RFs was detected only in Site3 (Fig. 4). A previous study reported that less abundant T-RFs are sensitive to environmental changes compared to the core denitrifying bacteria in wetlands for swine wastewater treatment (Zhang et al., 2017). Remarkably, in our study, both the dominant and less abundant T-RFs were sensitive to primary succession. The most probable reason for the above phenomenon is that the community compositions of *nosZ* denitrifiers are strongly shaped by the environmental conditions, such as denitrifying enzyme activity, soil organic carbon and total nitrogen (Brankatschk et al., 2011; Kandeler et al., 2006; Zeng et al., 2016). Our findings agree with past studies that the distribution of denitrification microorganisms depend on the environment (Chen et al., 2012; Zhang et al., 2017; Foshtomi et al., 2018).

### Relationships between *nosZ* denitrifying bacterial communities and soil properties

Environmental factors can significantly affect both the denitrification processes and the functional genes involved in the microbial pathway, and factors such as carbon substrate availability, moisture, pH, and other edaphic nutrients play crucial roles (Wallenstein et al., 2006). Göransson, Venterink & Bääth (2011) suggested that SOC and N were the most limiting resources that influenced soil development and microbial activity in early soils. Furthermore, Wardle, Walker & Bardgett (2004) showed that SOC was the most limiting factor for microbial growth and activity in early, unvegetated, barren soils, and the N-limitation effect occurred as carbon accumulated in the early soil. However, there is still no consensus on the major limiting factors of primary succession. Zeng et al. (2015) indicated that soil microbial processes and enzyme activities were significantly correlated with pH, and available P and K. Tang et al. (2016) showed that phosphorus had a strong influence on the abundance of nitrogen-cycling microorganisms in forest plantations, with a strong positive correlation between soil available phosphorus and nitrogen cycling-related microbial functional gene abundance. Knelman et al. (2012) showed that soil nutrient and carbon pools did not correlate with bacterial community composition, and pH was the only soil parameter that correlated with bacterial community composition. Overall, there is a close relationship between microorganisms and soil parameters, and because microorganisms are near the bottom of the food chain, changes in microbial community are often a precursor to changes in environmental parameters as a whole. Therefore, they are typically the first organisms to react to chemical and physical changes in the environment (Zeng et al., 2015). We found that soil pH, denitrifying enzyme activity, and available phosphorus and potassium significantly affected the composition of *nosZ* denitrifying bacterial communities. Soil pH was negatively correlated with the evenness of denitrifying bacteria, while soil available phosphorus and potassium were positively correlated with the Shannon–Weiner index. Soil available phosphorus and potassium, and denitrifying enzyme activity were positively linked to richness. Rangwala & Miller (2012) indicated that soil phosphorus limits plant productivity and indirectly affects soil microbial communities. Jha et al. (2017) found that *nosZ* community structure had the strongest correlation with soil moisture content and available phosphorus. Those results were in agreement with our findings showing the relationship between *nosZ-* denitrifiers and soil properties. While we focused specifically on *nosZ* abundance, previous studies have indicated that *nirS* and *nirK* communities are also strongly structured along gradients of soil water and phosphorus contents (Jha et al., 2017).

## Conclusions

We found that the variation in soil properties, denitrifying enzyme activity, abundance, and composition of the *nosZ* denitrifying bacterial community differed along successional stages. Soil pH decreased gradually in the course of succession, while SOC and TN significantly increased with the primary succession, and AP and AK increased gradually and peaked at Site3.The diversity and abundance of *nosZ*-denitrifiers was higher in successional 20–60 years soils compared to successional 0–5 years soils, and was highest at Site3 (40 years). Overall, 21 *nosZ* T-RFs were detected in the primary succession stages, but the dominant and specific T-RFs allowed for the identification to the genus level along a glacier forefield chronosequence. Of the fragments that were found, 90 bp T-RFs was consistent with the T-RF of *Azospirillum*, and 95 bp T-RFs was consistent with *Rubrivivax*, which were dominanting the soil in the early successional stages (0–20 years) and in the mature phase (40–60 years), respectively. Furthermore, the T-RFs of 100 and 275 bp were detected only in Site3 and Site4, and were consistent with *Bradyrhizobium* and *Pseudomonas*, respectively. Moreover, the unidentified 175 bp T-RFs was detected only in Site3. Of the measured abiotic factors, AP, AK and DEA correlated significantly with the abundance (*P* < 0.05 by Monte Carlo permutation test within RDA analysis). The results of this study provide the basis for predicting the changes in soil denitrifying bacterial community in the primary succession of Hailuogou Glacier retreat areas.

##  Supplemental Information

10.7717/peerj.7356/supp-1 Dataset S1 Soil properties in different soil successional stages SOC: Soil organic carbon; TN: Total nitrogen; AP: Available phosphorus; AK: Available potassium. DEA: Denitrifying enzyme activity. Site1: Soil successional ages ≤5 years; Site2: Soil successional ages ≤20 years; Site3: Soil successional ages ≤40 years; Site4: Soil successional ages ≤60 years.

10.7717/peerj.7356/supp-2 Dataset S2 The *nosZ* copy numbers of different soil successional stages Site1: Soil successional ages ≤5 years; Site2: Soil successional ages ≤20 years; Site3: Soil successional ages ≤40 years; Site4: Soil successional ages ≤60 years.

10.7717/peerj.7356/supp-3 Dataset S3 The *nosZ* gene diversity along successional stages Site1: Soil successional ages ≤5 years; Site2: Soil successional ages ≤20 years; Site3: Soil successional ages ≤40 years; Site4: Soil successional ages ≤60 years.

10.7717/peerj.7356/supp-4 Dataset S4 Pearson’s correlations between soil properties, denitrifying enzyme activity, and *nosZ* gene diversity.

10.7717/peerj.7356/supp-5 Dataset S5 The community composition of *nosZ*-denitrifiers at different soil successional stages Site1: Soil successional ages ≤5 years; Site2: Soil successional ages ≤20 years; Site3: Soil successional ages ≤40 years; Site4: Soil successional ages ≤60 years.

10.7717/peerj.7356/supp-6 Dataset S6 Redundancy analysis ordination diagram of the *nosZ*-denitrifying bacterial community composition associated with environmental variables SOC: Soil organic carbon; TN: Total nitrogen; AP: Available phosphorus; AK: Available potassium. DEA: Denitrifying enzyme activity. Site1: Soil successional ages ≤5 years; Site2: Soil successional ages ≤20 years; Site3: Soil successional ages ≤40 years; Site4: Soil successional ages ≤60 years.

## Additional Information and Declarations

### Data Availability

The following information was supplied regarding data availability: Raw data is available in the Supplemental Files and at GenBank: KT329435.
